# Ventilatory efficiency in combination with peak oxygen uptake improves risk stratification in patients undergoing lobectomy

**DOI:** 10.1016/j.xjon.2022.06.018

**Published:** 2022-07-03

**Authors:** Karolina Kristenson, Johan Hylander, Miklos Boros, Anna Fyrenius, Kristofer Hedman

**Affiliations:** a Departments of Anesthesiology and Intensive Care in Linköping and Biomedical and Clinical Sciences, Linköping University, Linköping, Sweden; b Departments of Thoracic and Vascular Surgery in Östergötland and Health, Medicine and Caring Sciences, Linköping University, Linköping, Sweden; c Departments of Respiratory Medicine in Linköping and Biomedical and Clinical Sciences, Linköping University, Linköping, Sweden; d Departments of Clinical Physiology in Linköping and Health, Medicine and Caring Sciences, Linköping University, Linköping, Sweden

**Keywords:** cardiopulmonary exercise testing, guidelines, lung cancer, peak oxygen uptake, risk stratification, ventilatory efficiency, CPET, cardiopulmonary exercise testing, DLCOc, carbon monoxide lung diffusion capacity corrected for hemoglobin, EqCo_2_ nadir, the lowest value (nadir) of the ventilation/VCO_2_ ratio during exercise, FEV1, forced expiratory volume in 1 second, MITS, minimally invasive thoracic surgery, MCPC, major cardiopulmonary complications, MPC, major pulmonary complications, VCo_2_, carbon dioxide elimination, VE, minute ventilation, VE/VCo_2_-slope, the slope of the increase in minute ventilation in relation to carbon dioxide output, Vo_2peak_, peak oxygen uptake

## Abstract

**Objective:**

We aimed to evaluate whether or not using the slope of the increase in minute ventilation in relation to carbon dioxide (VE/VCo_2_-slope), with a cutoff value of 35, could improve risk stratification for major pulmonary complications or death following lobectomy in lung cancer patients at moderate risk (Vo_2peak_ = 10-20 mL/kg/min).

**Methods:**

Single center, retrospective analysis of 146 patients with lung cancer who underwent lobectomy and preoperative cardiopulmonary exercise testing in 2008-2020. The main outcome was any major pulmonary complication or death within 30 days of surgery. Patients were categorized based on their preoperative cardiopulmonary exercise testing as: low-risk group, peak oxygen uptake >20 mL/kg/min; low-moderate risk, peak oxygen uptake 10 to 20 mL/kg/min and VE/VCo_2_-slope <35; and moderate-high risk, peak oxygen uptake 10 to 20 mL/kg/min and VE/VCo_2_-slope ≥35. The frequency of complications between groups was compared using χ^2^ test. Logistic regression was used to calculate the odds ratio with 95% CI for the main outcome based on the cardiopulmonary exercise testing group.

**Results:**

Overall, 25 patients (17%) experienced a major pulmonary complication or died (2 deaths). The frequency of complications differed between the cardiopulmonary exercise testing groups: 29%, 13%, and 8% in the moderate-high, low-moderate, and low-risk group, respectively (*P* = .023). Using the low-risk group as reference, the adjusted odds ratio for the low-moderate risk group was 3.44 (95% CI, 0.66-17.90), whereas the odds ratio for the moderate-high risk group was 8.87 (95% CI, 1.86-42.39).

**Conclusions:**

Using the VE/VCo_2_-slope with a cutoff value of 35 improved risk stratification for major pulmonary complications following lobectomy in lung cancer patients with moderate risk based on a peak oxygen uptake of 10 to 20 mL/kg/min. This suggests that the VE/VCo_2_-slope can be used for preoperative risk evaluation in lung cancer lobectomy.

## Graphical abstract

**Figure:**
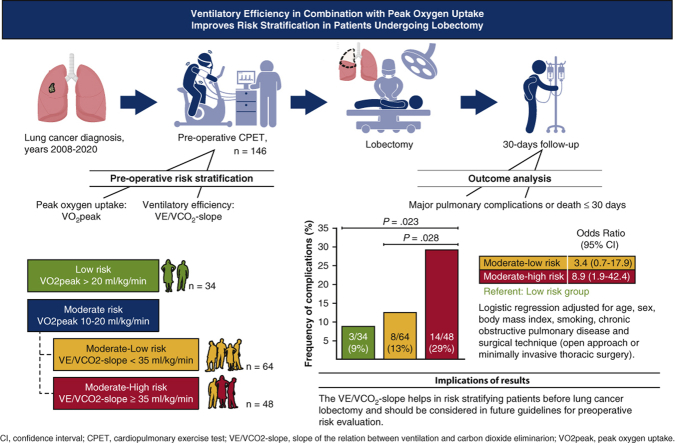

**Figure:**
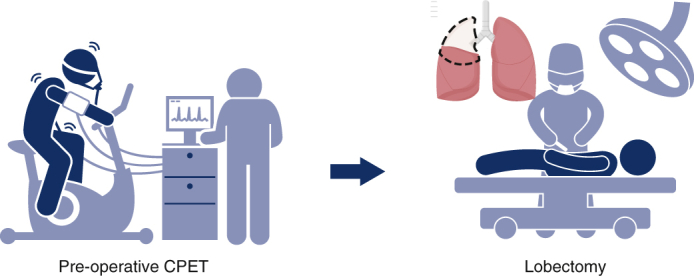
Preoperative cardiopulmonary exercise testing and complications following cancer lobectomy.

**Central Message**

Using the VE/VCo_2_-slope improves risk stratification for major pulmonary complications or death following lobectomy in lung cancer patients with moderate risk based on a Vo_2peak_ of 10 to 20 mL/kg/min.

**Perspective**

The present study highlights that preoperative risk assessment in patients with lung cancer can be improved if the VE/VCo_2_-slope with a cutoff value of 35 is considered in adjunct to Vo_2peak_. This suggests that the VE/VCo_2_-slope obtained from preoperative cardiopulmonary exercise testing should be considered in future guidelines for preoperative risk evaluation in lung cancer lobectomy.

As stated by the American Association for Thoracic Surgery expert panel consensus statement recently, defining which patients are at high risk for complications with lobectomy for lung cancer is challenging, but critical.^1^ Cardiopulmonary exercise testing (CPET) is considered the gold standard for the functional assessment and risk stratification of candidates for major pulmonary resection.^2^ Current international guidelines identify patients with an absolute peak oxygen consumption (Vo_2peak_) <10 mL/kg/min as high risk, patients with Vo_2peak_ 10 to 20 mL/kg/min as moderate risk, and those with Vo_2peak_ >20 mL/kg/min as low risk of perioperative complications or death.^3^^,^^4^ However, more recent studies have shown that patients considered at moderate risk still have a clinically relevant risk of morbidity and mortality after major anatomic pulmonary resection,^5^ which stresses the need for methods to further risk stratify this group of patients.

In addition to exercise capacity, CPET provides measures of ventilatory efficiency such as the slope of the increase in minute ventilation (VE) in relation to carbon dioxide output (the VE/VCo_2_-slope) or the lowest value (nadir) of the ventilation/VCo_2_ ratio during exercise (EqCo_2_ nadir). Both have been associated with negative events in pulmonary arterial hypertension and heart failure.^6^, ^7^, ^8^ During the past decade, studies on preoperative evaluation for lung surgery have found an association between the VE/VCo_2_-slope and both mortality^9^, ^10^, ^11^, ^12^ and perioperative pulmonary complications.^12^, ^13^, ^14^

An algorithm for preoperative stratification of patients’ risk of perioperative complications has been proposed, that incorporates both Vo_2peak_ and ventilatory efficiency.^2^ Patients in the moderate risk group (Vo_2peak_ = 10-20 mL/kg/min), are suggested to be further risk stratified into low-moderate or moderate-high groups, based on a VE/VCo_2_-slope less than or more than 35, respectively. Although the use of both the Vo_2peak_ and VE/VCo_2_-slope for risk stratification has been implemented in a few recent national guidelines,^15^^,^^16^ this approach remains to be validated.

We aimed to evaluate whether using the VE/VCo_2_-slope, with a cutoff value of 35, could improve risk stratification for major pulmonary complications (MPC) or death (primary outcome) or major cardiopulmonary complications (MCPC) or death (secondary outcome) following lobectomy in lung cancer patients at moderate risk (Vo_2peak_ of 10-20 mL/kg/min). We hypothesized that among patients in the moderate risk group, the frequency of complications would be higher for patients with a VE/VCo_2_-slope ≥35 compared with those with a VE/VCo_2_-slope <35.

## Material and Methods

### Participants

This was a single-center retrospective cohort study, including all patients with lung cancer who underwent lobectomy and a preoperative CPET during the years 2008 to 2020 at Linköping University Hospital, Linköping, Sweden. Ethical permission was granted by the Swedish Ethical Review Authority (Dnr 2020-03375, 2020-05284, 2021-00543). Informed consent was waived for this retrospective, de-identified analysis.

### Pulmonary Function Testing

As part of the preoperative evaluation, pulmonary function testing was performed either at the referring hospital (a minority of cases) or at the same center as the CPET. In the former case, results regarding pulmonary function were retrieved through medical records; in all other cases, raw data was available and analyzed. Data retrieved included static and dynamic lung volumes (forced expiratory volume in 1 second (FEV1), forced vital capacity, total lung capacity, and residual volume), and carbon monoxide lung diffusion capacity corrected for hemoglobin, (DLCOc). Spirometry measures were expressed as crude values as well as percentages of predicted (pp).^17^^,^^18^

### CPET

A maximal CPET was performed on a cycle ergometer, including 5 minutes of warm-up at 10 to 50 watts (W), followed by a continuous incremental ramp protocol of 10 to 20 W/min (eBike Basic, GE Medical Systems, GmbH). The warm-up and incremental workloads were chosen individually, aiming to reach exhaustion after 8 to 12 minutes of exercise. Patients were monitored with echocardiograph (Marquette CASE 8000, GE Medical Systems), systolic blood pressure, rating of perceived exertion (19 RPE scale), chest pain, and dyspnea (Borg^19^ CR-10 scale).

Gas exchange and ventilatory variables were analyzed on a breath-by-breath basis (Jaeger Oxycon Pro or Vyntus CPX; Viasys Healthcare). The system was calibrated before each test. Oxygen uptake (Vo_2_), carbon dioxide elimination (VCo_2_) and VE were presented numerically as 10-second means, excluding breaths with the highest and lowest values. Vo_2peak_ was defined as the average of the 2 highest consecutive 10-second mean Vo_2_ intervals at or close to the end of the exercise and presented as body mass indexed values (mL/kg/min), as well as percent of predicted.^20^ Ventilatory variables (the VE/VCo_2_-slope and EqCo_2_ nadir) were manually measured using commercially available software (Sentry Suite 3.10; CareFusion GmbH). The VE/VCo_2_-slope was determined as the slope of the VE/VCo_2_-curve, confined to the linear part up until the respiratory compensation point.^21^ The EqCo_2_ nadir was defined as the lowest value of the ventilatory equivalent for carbon dioxide during exercise.^8^ The EqCo_2_ nadir was used as the measure of ventilatory efficiency only in cases where the VE/VCo_2_-slope was indeterminate.

Each patient was categorized into 1 of 3 groups based on their preoperative Vo_2peak_ and VE/VCo_2_-slope (or EqCo_2_ nadir) values: low-risk group, Vo_2peak_ >20 mL/kg/min (irrespective of the VE/VCo_2_-slope); low-moderate risk group, Vo_2peak_ 10 to 20 mL/kg/min and VE/VCo_2_-slope <35; moderate-high risk group, Vo_2peak_ 10 to 20 mL/kg/min and VE/VCo_2_-slope ≥35.

### Outcome Registration and Definitions

Our primary outcome was MPC or death within 30 days from surgery, where MPC was defined as any of pneumonia, pulmonary embolus, empyema, delayed extubation (not able to extubate in the operation room directly after surgery), reintubation, reoperation, acute respiratory distress syndrome, respiratory insufficiency, or pulmonary edema.

Our secondary end point was MCPC or death within 30 days from surgery, defined as any of the complications listed above (ie, MPC) or any of new onset arrhythmia, cerebral vascular insult, myocardial infarction, or acute renal failure.

Data from the CPET database was cross-linked with 3 separate databases, using the unique Swedish social security number, to ascertain full coverage on outcomes and comorbidities. First, the Swedish National Quality Register for General Thoracic Surgery^22^ was used to retrieve data on in-hospital complications, comorbidities, operation code and surgical technique (eg, open approach or minimally invasive thoracic surgery [MITS]). These data were then cross-linked with The Swedish National Patient Register, containing all inpatient and outpatient hospital diagnoses of each Swedish citizen.^23^ Finally, the survival status and date of death were determined in the Swedish Cause of Death Register, maintained at the National Board of Health and Welfare.^23^

Definitions of complications and comorbidities harmonize with recent international guidelines.^24^ The agreement in lung cancer diagnoses (C34 according to International Statistical Classification of Diseases and Related Health Problems–10th Revision) recorded in the 2 registers varied between 93% to 97% at the current surgical clinic at Linköping University Hospital between the years 2013 and 2019.^25^

### Reproducibility

Inter- and intrarater variability were determined for the VE/VCo_2_-slope and the EqCo_2_ nadir in 40 randomly selected measurements, by calculating the intraclass correlation coefficient, as well as the coefficient of variation.^26^

### Statistical Analysis

Cross-linking of databases and database management were performed using R Studio version 1.1.456 (R Foundation for Statistical Computing). Statistical analyses were performed with SPSS 23.0.0.2 (IBM-SPSS Inc). Between-group differences in mean values were compared with the independent *t* test. Proportions were compared with the χ^2^ test.

Logistic regression was used to determine the odds ratio (OR) with a 95% CI for the primary and secondary outcomes, based on the preoperative CPET group. Analyses were performed unadjusted as well as adjusted for: chronic obstructive pulmonary disease (preoperative diagnosis in The Swedish National Patient Register^23^), age, sex, body mass index, smoking (according to status at the preoperative CPET), and surgical technique (open approach or MITS).

We performed 3 sensitivity analyses. First, to determine whether or not including only truly maximal exercise tests would influence our results, we excluded patients with a respiratory exchange ratio <1.05 in combination with either <85% of predicted maximal heart rate (and without beta-blocker medication) or a breathing reserve >30%. Second, categorizing patients with both ppFEV1 and ppDLCO >80% as low risk subjects, regardless of results from CPET (in accordance with guidelines from the European Respiratory Society and European Society of Thoracic Surgeons^3^). Third, including data only from years 2017 to 2020, to increase the proportion of patients undergoing MITS.

## Results

### Patient Characteristics

**Figure 1:**
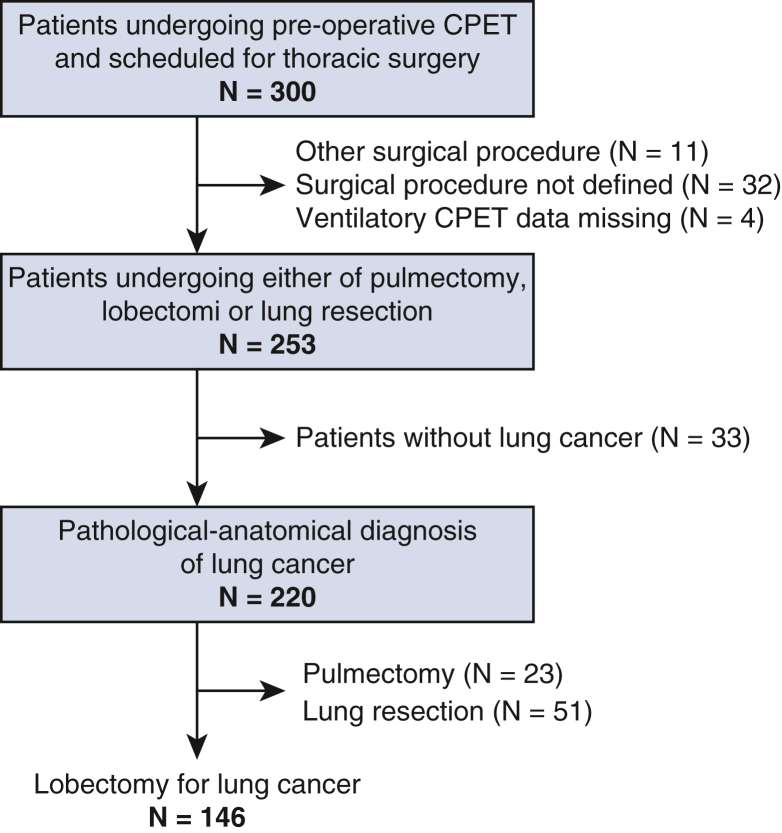
Study flowchart. *CPET*, Cardiopulmonary exercise testing.

A total of 146 patients (82 women [56%]; mean age, 71 ± 8 years) with a pathological-anatomical diagnosis of lung cancer who had undergone lobectomy (including bilobectomy, n = 10) were included (Figure 1). Two patients (1.4%) died within 30 days of the operation (both men and in the moderate-high risk group). In total, 25 patients (17%) experienced an MPC or died and 35 patients (24%) experienced an MCPC or died. Patient characteristics are presented in Table 1. Open approach and MITS techniques were used in 131 (90%) versus 15 (10%) of patients, respectively. No difference was found in complication frequency based on these 2 surgical techniques (for MPC, *P* [χ^2^] = .76; for MPCP, *P* [χ^2^] = .70). Overall, male patients more often experienced complications than female patients (MPC, 28% vs 9% [*P* = .004] and MCPC, 39% vs 12%; *P* < .001).

**Table 1:** Patient characteristics by occurrence of major pulmonary complications (MPCs) or death within 30 days of lobectomy

Variable	All patients (N = 146)	MPC or death (n = 25)	No MPC or death (n = 121)	*P* value
Basic characteristic				
Age (y)	71 ± 8	70 ± 7	71 ± 8	.439
Height (cm)	168 ± 9	171 ± 8	168 ± 9	.115
Weight (kg)	75 ± 16	75 ± 13	76 ± 17	.821
BMI	27 ± 5	26 ± 4	27 ± 5	.273
Spirometry				
FEV1 (L/min)	2.1 ± 0.6	2.2 ± 0.6	2.1 ± 0.6	.364
ppFEV1 (%)	77 ± 20	75 ± 19	78 ± 20	.523
VC (L)	3.3 ± 0.8	3.5 ± 0.8	3.3 ± 0.8	.357
ppFVC (%)	72 ± 22	79 ± 20	70.2 ± 21.9	.061
FEV1/VC	0.6 ± 0.1	0.6 ± 0.1	0.6 ± 0.1	.741
DLCOc (mmol/min/kPa)	5.5 ± 1.7	5.2 ± 1.2	5.6 ± 1.8	.374
ppDLCOc (%)	78 ± 20	68.3 ± 16.0	80 ± 20	.011
TLC (L)	6.1 ± 1.2	6.3 ± 1.2	6.0 ± 1.2	.350
ppTLC (%)	99 ± 15	93 ± 16	100 ± 15	.073
RV (L)	2.7 ± 0.7	2.7 ± 0.7	2.7 ± 0.8	.954
ppRV (%)	114 ± 35	110 ± 29	115 ± 36	.494
Cardiopulmonary exercise test				
Watt_peak_	90.7 ± 32.6	85.5 ± 28.8	91.8 ± 33.4	.380
ppWatt_peak_ (%)	69 ± 19	61 ± 18	70.8 ± 19.2	.018
Vo_2peak_ (mL/kg/min)	17.4 ± 3.8	16.8 ± 3.6	17.5 ± 3.8	.427
ppVo_2peak_ (%)	81 ± 15	72 ± 12	83 ± 15	<.001
VE/VCo_2_-slope	34.0 ± 6.4	38.1 ± 7.2	33.2 ± 5.9	.001
EqCo_2_ nadir	33.0 ± 5.2	36.3 ± 5.8	32.3 ± 4.8	<.001

Values are presented as mean ± SD. *BMI*, Body mass index; *FEV1*, forced expiratory volume in 1 second; *pp*, percent of predicted; *VC*, vital capacity; *FVC*, forced vital capacity; *DLCOc*, diffusing capacity of the lungs for carbon monoxide, corrected for hemoglobin; *TLC*, total lung capacity; *RV*, residual volume; *V**o*_*2peak*_, peak oxygen uptake; *VE*, minute ventilation; *VC**o*_*2*_, carbon dioxide elimination; *EqC**o*_*2*_, ventilatory equivalent for carbon dioxide.

FEV1 and forced vital capacity were registered in all patients, whereas DLCOc was missing in 21 patients (14%). Patients experiencing MPC or death had no significant difference in mean ppFEV1, but lower mean ppDLCOc compared with patients without a negative primary outcome (Table 1). Mean VE/VCo_2_-slope and EqCo_2_ nadir were higher and the mean ppVo_2peak_ and maximal workload (ppWatt_peak_) were lower in the group with MPC or death. There was no difference in mean weight-indexed Vo_2peak_ between groups.

### CPET

Inter- and intrarater agreement for measures of the VE/VCo_2_-slope and the EqCo_2_ nadir was high (Table E1). In 11 (7.5%) patients, the VE/VCo_2_-slope was not possible to measure, and the categorization into CPET groups was in these cases based on the EqCo_2_ nadir. Out of the 146 patients, 34 (23%) were categorized as low risk, 64 (44%) as low-moderate risk, and 48 (33%) as moderate-high risk. No patient who underwent pulmonary lobectomy had a Vo_2peak_ <10 mL/kg/min. Table 2 shows the distribution of comorbidities across the CPET groups.

**Table 2:** Distribution of comorbidities across groups defined by preoperative cardiopulmonary exercise testing peak oxygen uptake and ventilatory efficiency

Variable	Moderate-high risk∗ (n = 48)	Low-moderate risk∗ (n = 64)	Low risk∗ (n = 32)	Total (N = 146)
Coronary artery disease	5 (10)	6 (9)	0 (0)	11 (8)
Previous cardiac surgery	8 (17)	11 (17)	3 (9)	22 (15)
Previous cerebrovascular insult	4 (8)	6 (9)	0 (0)	10 (7)
Current treatment for heart failure	7 (15)	7 (11)	0 (0)	14 (10)
Current treatment for hypertension	21 (44)	26 (41)	10 (29)	57 (39)
Current treatment for arrhythmia	6 (13)	8 (13)	1 (3)	15 (10)
Diabetes mellitus	7 (15)	7 (11)	1 (3)	15 (10)
Chronic kidney disease	4 (8)	6 (9)	0 (0)	10 (7)
Chronic obstructive pulmonary disease	33 (48)	41 (44)	17 (29)	91 (41)
Body mass index >35	3 (6)	5 (8)	0 (0)	8 (5)
Smoking				
Never	2 (4)	8 (13)	7 (21)	17 (12)
Current	30 (63)	37 (58)	16 (47)	83 (57)
Previous	16 (33)	19 (30)	11 (32)	46 (32)

Values are presented as n (%).

∗ Moderate-high and moderate-low risk defined as a peak oxygen uptake of 10 to 20 mL/kg/min and a slope of the increase in minute ventilation in relation to carbon dioxide output ≥35 or <35, respectively; low risk defined as a peak oxygen uptake >20 mL/kg/min with any slope of the increase in minute ventilation in relation to carbon dioxide output value.

Major pulmonary complications or death was twice as common in patients with a VE/VCo2-slope ≥35 than in patients with a VE/VCo2-slope <35 (26% vs 12%, respectively; rate ratio [RR], 2.22; *P* = .030). When also including cardiac complications (ie, MCPC), a VE/VCo2-slope ≥35 or <35 was not discriminative for MCPC or death (29% vs 20%, respectively; RR, 1.51; *P* = .19). In contrast to the VE/VCo2-slope, having a Vo_2peak_ <20 mL/kg/min compared with ≥20 mL/kg/min was not discriminative for MPC or death (20% vs 9%, respectively; RR, 2.22, *P* = .14) but it was discriminative for MCPC or death (29% vs 9%, respectively; RR, 3.22, *P* = .018).

**Figure 2:**
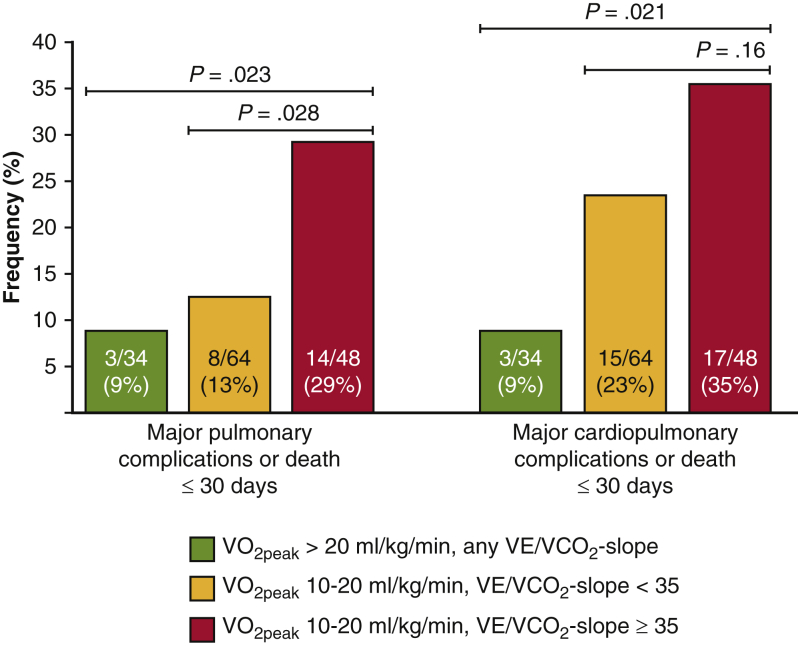
Frequency of complications across groups defined by preoperative cardiopulmonary exercise testing peak oxygen uptake and ventilatory efficiency. *V**o*_*2peak*_, Peak oxygen uptake; *VE*, minute ventilation; *VC**o*_*2*_, carbon dioxide elimination.

The frequency of complications differed between the three CPET groups, both for MPC or death (*P* = .023) and for MCPC or death (*P* = .021) (Figure 2). A statistically significant difference was found between the moderate-high and low-moderate risk group for MPC or death (29% vs 13%; *P* = .028) but not for MCPC or death (35% vs 23%; *P* = .16). Female and male patients had similar relative risk differences between different CPET risk groups, but male patients reached higher absolute values in complication frequencies (Table E2).

The unadjusted and adjusted ORs of experiencing an MPC or death and MCPC or death according to CPET group are presented in Table 3. Three sensitivity analyses were performed for our main outcome (MPC or death within 30 days), where the unadjusted and adjusted OR for being in the moderate-high risk group (reference: low-risk group) were calculated. First, when excluding 24 patients with a suspected nonmaximal CPET, the unadjusted OR was 4.30 (95% CI, 1.07-17.39) and adjusted OR 8.59 (95% CI, 1.63-45.28), respectively. Second, 26 subjects with both ppFEV1 and ppDLCO >80% were recategorized from the moderate-high (n = 7) or low-moderate (n = 16), or previously noncategorizable due to Vo_2peak_ < 20 mL/kg/min but missing data on ventilatory efficiency (n = 3), into the low-risk group (regardless of results from CPET). Using this new categorization, logistic regression revealed an unadjusted OR of 3.52 (95% CI, 1.26-9.81), and an adjusted OR of 3.98 (95% CI, 1.18-13.39), respectively, for being in the moderate-high risk group. Third, when including data only from years 2017 to 2020 (n = 77), The proportion of MITS increased to 20% and revealed an unadjusted OR of 10.50 (95% CI, 1.19-92.72), and an adjusted OR of 35.57 (95% CI, 2.23-567.90), respectively.

**Table 3:** Unadjusted and adjusted odds ratios (95% CI) for postoperative complications or death following cancer lobectomy based on preoperative risk determined by cardiopulmonary exercise testing (CPET)

Variable	Unadjusted analysis	Adjusted analysis∗
Major pulmonary complications or death		
CPET low risk†	1.00 (Referent)	1.00 (Referent)
CPET low-moderate risk‡	1.48 (0.37-5.97)	3.44 (0.66-17.90)
CPET moderate-high risk§	4.26 (1.12-16.23)	8.87 (1.86-42.39)
Major cardiopulmonary complications or death		
CPET low risk†	1.00 (Referent)	1.00 (Referent)
CPET low-moderate risk‡	3.16 (0.85-11.83)	6.66 (1.42-31.23)
CPET moderate-high risk§	5.67 (1.51-21.31)	11.78 (2.55-54.34)

Values are presented as odds ratio (95% CI).

∗ Included covariates were chronic obstructive pulmonary disease, age, sex, body mass index, smoking, and surgical technique (ie, open approach or minimally invasive thoracic surgery).

† CPET low risk = peak oxygen uptake >20 mL/kg/min.

‡ CPET low-moderate risk = peak oxygen uptake 10 to 20 mL/kg/min and slope of the increase in minute ventilation in relation to carbon dioxide output ≤35.

§ CPET moderate-high risk = peak oxygen uptake 10 to 20 mL/kg/min and slope of the increase in minute ventilation in relation to carbon dioxide output >35.

## Discussion

**Figure 3:**
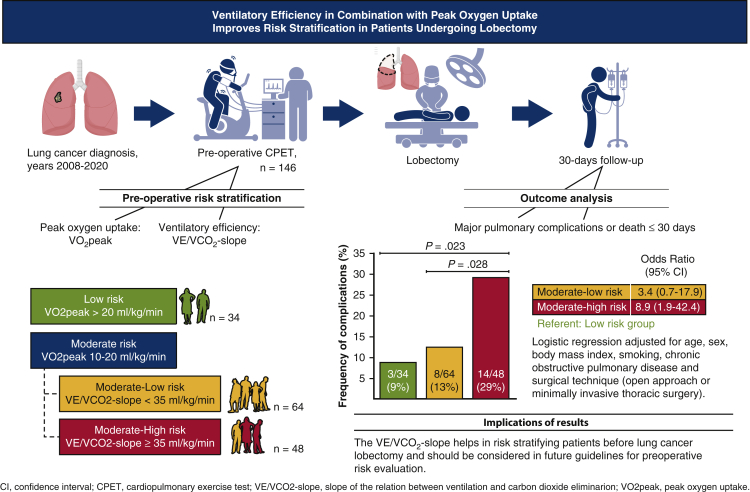
Study methods, results, and implications.

The main finding of this retrospective cohort study was that using the VE/VCo_2_-slope in addition to Vo_2peak_ improved risk stratification in patients with lung cancer undergoing lobectomy (Figure 3). Specifically, in patients with a Vo_2peak_ 10 to 20 mL/kg/min, defined as at moderate risk by international guidelines,^3^^,^^4^ major pulmonary complications were twice as common in subjects with a VE/VCo_2_-slope ≥35.

This finding is important because there is an unmet need to further risk stratify patients with a moderate preoperative risk, based on Vo_2peak_. This group of patients is large, heterogeneous and at a non-negligible risk of suffering complications. A recent study based on the European Society of Thoracic Surgeons’ database included patients with reduced lung function with available CPET data.^5^ They found that 72% of patients belonged to the moderate risk group. Similar data was found in our study where as many as 77% of patients who underwent lobectomy had a Vo_2peak_ between 10 and 20 mL/kg/min.

We found that the proposed cutoff value for the VE/VCo_2_-slope ≥35 could risk discriminate for MPC, whereas the proposed cutoff value for Vo_2peak_ (>20 mL/kg/min) identified subjects at risk of MCPC.^2^ These findings, with an association between ventilatory inefficiency and pulmonary complications, but not major cardiopulmonary complications, harmonize with results from previous cohorts.^9^^,^^12^ This may be taken as an additional argument to include both parameters in a risk stratification algorithm because they are partially associated with different types of complications. The aim with this study was to validate a CPET categorization algorithm already implemented in some national guidelines. However, it is possible that other cutoff values than those presented in this study are even more discriminative for postoperative negative events.

In the current study, compared with patients with a Vo_2peak_ >20 mL/kg/min (low risk), patients with a Vo_2peak_ of 10 to 20 mL/kg/min and with VE/VCo_2_-slope values ≥ 35 (moderate-high risk) had an adjusted OR for MPC or death of 8.87 (95% CI, 1.86-42.39), versus 11.78 (95% CI, 2.55-54.34) for MCPC or death, respectively. When comparing the 2 moderate-risk groups (excluding subjects with a Vo_2peak_ >20 mL/kg/min), the complication frequency was significantly higher in the moderate-high risk versus the low-moderate risk group for MPC, but not for MCPC. This further underscores the association between ventilatory inefficiency and the risk of pulmonary complications. This association is physiologically reasonable because ventilatory inefficiency measured from CPET in clinical practice can be viewed as a noninvasive measurement of dead space ventilation. Therefore, it is logical to assume that ventilatory inefficiency is associated with respiratory frailty. Although patients in the moderate-high risk group were found to have an increased risk of postoperative complications, they should not per definition be excluded from surgical lobectomy. However, these patients should be evaluated with caution in relation to other comorbidities, frailty, and patient preference in the decision to perform lobectomy, sublobar resection, or stereotactic radiation. When performing lobectomy in these patients, it is of great importance to involve an experienced surgeon, intensify physical therapy, and optimize pain treatment to promote early mobilization.

Although most studies on ventilatory efficiency in the literature on preoperative evaluation have used the VE/VCo_2_-slope as their primary ventilatory parameter, the EqCo_2_ nadir has been shown to provide greater prognostic value than the VE/VCo_2_-slope in patients with suspected heart failure.^8^ The VE/VCo_2_-slope has also been found less reproducible than the EqCo_2_-nadir in sequential testing,^27^ which was replicated in our measures of reproducibility (Table E1). In our study, the EqCo_2_ nadir was used as a parameter of ventilatory efficiency only when it was not possible to measure the VE/VCo_2_-slope, which occurred in <10% of cases, and there is a known close correlation between the VE/VCo_2_-slope and the EqCo_2_-nadir.^27^ Considering the advantage in terms of intrarater variability, the feasibility in measurement and the prior results in cardiac patients, the prognostic implications of using the EqCo_2_-nadir in preference to the VE/VCo_2_-slope in preoperative evaluation should be determined in future studies.

In a sensitivity analysis, we excluded subjects with a potentially submaximal CPET, based on the respiratory exchange ratio, breathing reserve, and maximal heart rate. This approach yielded very similar results as the primary analysis, which implies that a maximal exercise effort is not mandatory for the algorithm to be valid. This is logical because per definition, the VE/VCo_2_-slope is measured at a submaximal exercise level (ie, below the ventilatory compensation point).^21^

Notably, we found male gender to be a significant risk factor for both MPC and MCPC (RR, 3.37 and 3.15, respectively). This finding is inconsistent with several previous studies.^5^^,^^10^, ^11^, ^12^ However, in these studies a majority of included patients were men, potentially leading to a relative loss in power to detect a true risk difference. In the current study, the proportion of men was roughly 50%. In the work of Miyazaki and colleagues,^9^ 42% of included patients were women, and they found a 9-fold increase in 90-day mortality for men compared with women. Moreover, recent data from a national Swedish cohort found female gender to be associated with better survival following pulmonary resection for lung cancer, regardless of comorbidities, socioeconomic status, lifestyle factors, type and extent of surgery, tumor characteristics, and stage of disease.^28^ The reason for this increased occurrence of negative events among men after major anatomic pulmonary resection is unknown. In this study, both male and female patients had similar difference in relative risk for complications between the CPET risk groups. However, due to the male patients’ higher overall complication rates, male patients in the moderate-high risk group reached a complication frequency of 50% for MPC compared with 14% among women, which is a substantial both in relative and absolute terms. However, adjusting our multivariate models for gender and baseline risk factors did not change the statistical significance for the CPET group being associated with MPC and MCPC.

This study has some limitations. First, as a single-center study, the results are not necessarily generalizable to other settings and centers. Nevertheless, including all subjects over a period of 12 years generated a comparably large population, and the basic cohort characteristics harmonize well with previously published national data.^16^ Secondly, the retrospective approach excluded the possibility of prospectively recording complications. However, we used 2 Swedish registries of known high quality to define the occurrence of complications, and because only major complications were included as outcome, the risk of underreporting in the registries should be low. Third, we were unable to include patients with a very high risk of complications (Vo_2peak_ <10 mL/kg/min) because these patients did not undergo surgery at our center. Although this implies that exercise capacity is already an important part of the preoperative, multidisciplinary decision making in these patients, it would be of value to compare the risk of complications in this group with the moderate risk groups. However, given their very high risk of complications, this comparison would require particular ethical considerations. Fourth, international guidelines currently suggest referral to CPET in patients with nonoptimal spirometry data, in part based on the limited availability of CPET at many centers. In this study, all patients who underwent lobectomy for lung cancer with preoperative CPET were included, thereby potentially including a healthier population. Therefore, we performed a sensitivity analysis where only patients with abnormal results from spirometry were included in the moderate risk group. Although the overall difference in risk of MPC between groups was similar when spirometry data were considered, the greatest point estimates in OR between moderate-high and low risk group were found in the original analysis. This suggests that results from CPET are more important than spirometry results in risk stratification. Finally, this dataset included a fairly low proportion of MITS. However, when only including data from years 2017 to 2020 the proportion of MITS procedures increased to 20%, whereas our main findings persisted and point estimates for OR between risk groups increased, implying that the current results are valid also in a contemporary setting.

## Conclusions

Incorporation of the VE/VCo_2_-slope in preoperative CPET algorithms using a cutoff value of 35, can improve risk stratification in patients with moderate risk (Vo_2peak_ = 10-20 mL/kg/min). These results suggest that this parameter of ventilatory efficiency is associated with major pulmonary complications after lung cancer lobectomy. When replicated in other cohorts, incorporation of the VE/VCo_2_-slope could be considered in future guidelines for preoperative risk evaluation in lung cancer lobectomy.

### Conflict of Interest Statement

The authors reported no conflicts of interest.

The *Journal* style requires editors and reviewers to disclose conflicts of interest and to decline handling or reviewing manuscripts for which they have a conflict of interest. The editors and reviewers of this article have no conflicts of interest.

## Figures and Tables

**Table E1:** Reproducibility of study measurements

	Rater A	Rater B	Intrarater		Interrater	
	Mean ± SD	Mean ± SD	COV (%)	ICC (95% CI)∗	COV (%)	ICC (95% CI)∗
VE/VCo_2_-slope	33.7 ± 6.7	33.7 ± 7.3	5.2	0.94 (0.88-0.97)	7.0	0.90 (0.82-0.95)
EqCo_2_ nadir	33.7 ± 5.8	33.7 ± 5.6	2.1	0.98 (0.97-0.99)	2.8	0.97 (0.95-0.99)

*COV*, Coefficient of variance in percent (determined by the S_method_); *ICC*, intraclass correlation coefficient; *VE*, minute ventilation; *VC**o*_*2*_, carbon dioxide elimination; *EqC**o*_*2*_, ventilatory equivalent for carbon dioxide.

∗ Single measures, two-way mixed absolute agreement ICC.

**Table E2:** Number and proportion of patients with complications stratified by sex and cardiopulmonary exercise test group

Variable	MPC			MCPC		
	Male	Female	*P* value	Male	Female	*P* value
Low-risk group	3 (17)	0 (0)	.23	3 (17)	0 (0)	.23
Low-moderate risk group	5 (19)	3 (8)	.25	10 (40)	5 (13)	.034
Moderate-high risk group	10 (50)	4 (14)	.011	12 (60)	5 (18)	.005
Overall	18 (28)	7 (9)	.004	25 (39)	10 (12)	<.001

Values are presented as n (%). *MPC*, Major pulmonary complications or death within 30 days of surgery; *MCPC*, major cardiopulmonary complications or death within 30 days of surgery.
